# Front and hind paw differential analgesic effects of amitriptyline, gabapentin, ibuprofen, and URB937 on mechanical and cold sensitivity in cisplatin-induced neuropathy

**DOI:** 10.1177/1744806919874192

**Published:** 2019-09-23

**Authors:** Jeremy M Thompson, Henry L Blanton, Agata Pietrzak, William Little, Caitlyn Sherfey, Josée Guindon

**Affiliations:** 1 Department of Pharmacology and Neuroscience, Texas Tech University Health Sciences Center, Lubbock, TX, USA; 2 Center of Excellence for Translational Neuroscience and Therapeutics, Texas Tech University Health Sciences Center, Lubbock, TX, USA

**Keywords:** Front paws, hind paws, chemotherapy-induced neuropathy, amitriptyline, gabapentin, ibuprofen, URB937, mechanical and cold allodynia

## Abstract

Cisplatin is a widely used platinum-derived antineoplastic agent that frequently results in peripheral neuropathy. Therapeutic strategies for neuropathic pain are limited and characterized by variable efficacy and severe adverse effects. Clinical translation of novel analgesics has proven difficult with many agents demonstrating preclinical efficacy failing in clinical trials. Preclinical studies frequently assess pain behaviors in the hind paws; however, the front paws have a greater degree of the fine sensorimotor functions characteristically damaged by chemotherapy-induced neuropathy. This is the first study to assess pain responses in the front paws. Here, we test the hypothesis that mouse front paws exhibit pain-related alterations in mechanical and thermal (cold) sensitivity in a murine model of cisplatin-induced neuropathy and that pharmacological treatment with amitriptyline, gabapentin, ibuprofen, and URB937 normalize pain behaviors in the front and hind paws. Cold (acetone withdrawal latencies) and mechanical (von Frey withdrawal thresholds) sensitivity were significantly increased and decreased respectively in both the front and the hind paws following initiation of weekly systemic (intraperitoneal) cisplatin injections (5 mg/kg). For the hind paws, systemic administration of amitriptyline (30 mg/kg), gabapentin (100 mg/kg), ibuprofen (0–10 mg/kg), or URB937 (0–10 mg/kg) resulted in a decrease in acetone withdrawal latencies and increase in von Frey withdrawal thresholds with return to normal values at the highest doses tested. For the front paws, return to baseline values for the highest doses was found for cold allodynia but not mechanical allodynia, where the highest doses failed to return to baseline values. These results indicate that mouse front paws exhibit pain-related changes in cisplatin-induced neuropathy and that drug effects can vary based on testing stimulus and location. This suggests that front paw responses across multiple modalities provide reliable and accurate information about pain-related drug effects. Future studies should be aimed at elucidating the mechanisms underlying these differential effects.

## Introduction

Cisplatin is a platinum-derived antineoplastic drug that is on the World Health Organization Model List of Essential Medicines due to its efficacy at treating solid malignancies including ovarian, testicular, and head and neck cancers.^1^ It acts by crosslinking purine bases in DNA, thereby causing DNA damage and interfering with DNA repair mechanisms, leading to cellular apoptosis.^1^ Chemotherapy-induced peripheral neuropathy is a dose-dependent side effect of cisplatin therapy with significant associated morbidity.^1,2^

Chemotherapy-induced neuropathy preferentially affects large, thickly myelinated axons and presents in a “glove and stocking” distribution.^3,4^ Clinically, this presents as a sensory, motor, and/or autonomic neuropathy and includes symptoms such as mechanical/thermal sensitivity, altered sensory perception and touch, and impaired fine motor skills. Cisplatin-induced neuropathy in particular is associated with numbness, paresthesias, and mechanical and thermal sensitivity in approximately 92% of patients treated with cisplatin.^4^ Neuropathic symptoms are progressive and persist for several months, potentially resulting in a permanent peripheral neuropathy.

Treatment of this neuropathy is a clinical challenge due to limited treatment options, variable efficacy, and significant side effects.^5–7^ Addressing this clinical need has proven challenging as many agents that demonstrate preclinical analgesic effects often fail in clinical trials.^8,9^ Several explanations have been proposed to explain this effect, including methodological issues with assessment of pain behaviors in preclinical models.^10^ Pain behaviors are typically assessed on the plantar surface of the hind paws;^11^ however, this does not necessarily recapitulate the clinical picture of the pain experience in humans. In particular, the rodent front paws exhibit fine sensorimotor function that is characteristically damaged in cisplatin-induced neuropathy.^4,12^ Reponses of the front paws to the development and treatment of cisplatin-induced neuropathy have not yet been assessed.

This study investigates for the first time the effects of cisplatin-induced neuropathy on pain behaviors in the front and hind paws. We determined mechanical and thermal (cold) responses before and after development of cisplatin-induced neuropathy, as well as the effects of treatment with the peripherally restricted fatty acid amide hydrolase (FAAH) inhibitor URB937,^13,14^ the nonsteroidal anti-inflammatory drug (NSAID) ibuprofen, the antidepressant amitriptyline, and the anticonvulsant gabapentin. Important novelties of this study include determining pain-related changes in the mouse front paws as well as comparison of drug effects on mechanical and thermal sensitivity between the front and hind paws.

## Methods

### Animals

One hundred and twenty-four adult male C57BL/6 mice (28–35 g) were housed in a temperature-controlled room and maintained on a 12-h day/night cycle with unrestricted access to food and water. All animal care and experimental procedures used were approved and conducted in accordance with National Institutes of Health accepted guidelines^15^ and with approval from the Institutional Animal Care and Use Committee at Texas Tech University Health Sciences Center.

### Experimental protocol

Pain behaviors (see “Pain behaviors” subsection) were measured before and every 2 days for 28 days after beginning pain induction (see “Cisplatin-induced neuropathy pain model” subsection). Systemic drug effects were determined 28 days after beginning pain induction. Amitriptyline, gabapentin, ibuprofen, or URB937 was injected intraperitoneally (i.p.), and the same volume (1 ml/kg) was used for all injections. Behaviors were tested at 30 min and 150 min after drug injection. It has been previously established that intraperitoneal (i.p.) injections display similar pharmacokinetics to *per os* doses.^16^ Pharmacokinetic curves exist for all four drugs in murine models, and, in each case, the 30-min time point displays a peak concentration of drug in serum plasma.^17–20^ The 150-min time point was also evaluated to determine the prolonged antinociceptive effects of each drug while still maintaining a pharmacodynamically relevant serum plasma concentration.^17–20^

Front paws were evaluated prior to testing hind paws. Different groups of mice were used to test the effects of amitriptyline, gabapentin, ibuprofen and URB937. For the ibuprofen and URB937 doses tested (ranging between 0 and 10 mg/kg), each dose was evaluated in the dosing increment of the lowest to the highest dose with a 96-h interval between doses, and residual drug effect was absent demonstrated by values back to baseline levels after 96 h.

### Cisplatin-induced neuropathy pain model

Cisplatin chemotherapy-induced neuropathy was induced as described previously.^21–23^ Mice received i.p. injections of cisplatin (5 mg/kg) or saline (sham control) every seven days to induce neuropathy. Injections were done by diluting cisplatin in sterile 0.9% saline and injecting a volume of 10 mL/kg of body weight. Prior to cisplatin treatment, each mouse was treated subcutaneously with 1 mL of 4% sodium bicarbonate to prevent nephrotoxicity-induced lethality.^21^ Pain induction and stability were monitored by testing behaviors every 2 days for 28 days after the initial injection (see “Experimental protocol” subsection).

### Pain behaviors

*Mechanical withdrawal thresholds* were determined using an automated von Frey anesthesiometer applied to the plantar surface of the right and left front and hind paws.^21^ Prior to testing, mice were allowed to habituate for 30 min in individual plexiglass containment chambers placed on a wire mesh table. Testing was performed using a digital electronic von Frey anesthesiometer (IITC Life Sciences, Woodland Hills, CA, USA) equipped with a semiflexible plastic tip that was used to apply force to animal’s midplantar front and hind paws. Two replicates were obtained for each paw. No difference in response was observed between the right and left paws, so mechanical sensitivity was determined by averaging right- and left-sided responses.

*Cold responses* were determined using the acetone test by applying a drop (approximately 20 µL) of acetone to the plantar surface of the right and left front and hind paws.^21^ Prior to testing, mice were placed in individual plastic cages on an elevated platform and were habituated for at least 30 min until exploratory behaviors ceased. Acetone was loaded into a 1 mL syringe without a needle. Air bubbles were cleared from the syringe prior to acetone application. One drop of acetone was applied through the mesh platform onto the plantar surface of the paw. Time spent attending to the acetone-stimulated paw was measured over a 60-s observation period after acetone application was recorded. Three replicates were obtained for each paw. No difference in response was observed between right and left paws, so cold sensitivity was determined by averaging right- and left-sided responses.

### Drugs

Cisplatin was purchased from Tocris (Ellisville, MO, USA) and dissolved in normal saline (0.9% NaCl). Amitriptyline hydrochloride, gabapentin, and ibuprofen were purchased from Sigma-Aldrich (St. Louis, MO, USA) and dissolved in normal saline (0.9% NaCl). URB937 (3′-carbamoyl-6-hydroxy-[1,1′-biphenyl]-3-yl cyclohexylcarbamate) was purchased from Cayman Chemical (Ann Arbor, MI, USA) and dissolved in dimethylsulfoxide. Doses of amitriptyline, gabapentin, ibuprofen, and URB937 were selected based upon efficacy demonstrated in the previous studies.^21–23^

### Statistics

All experiments were conducted in a blinded manner. Animals were randomly assigned to experimental conditions. Pain behavior for each treatment group was expressed as mean ± standard error of the mean (SEM). Paw withdrawal thresholds (mechanical) and latencies (cold) were calculated for each paw and averaged. Repeated measures one- or two-way analysis of variance (ANOVA) with Bonferroni posttests were used where appropriate to determine significance. GraphPad Prism 8.0 (GraphPad Software, San Diego, CA, USA) and SPSS (version 25.0; SPSS Incorporated, Chicago, IL, USA) statistical software were used for analysis. Statistical significance was accepted at P < 0.05.

## Results

### Front relative to hind paws changes in cisplatin-induced mechanical sensitivity

**Figure 1.**
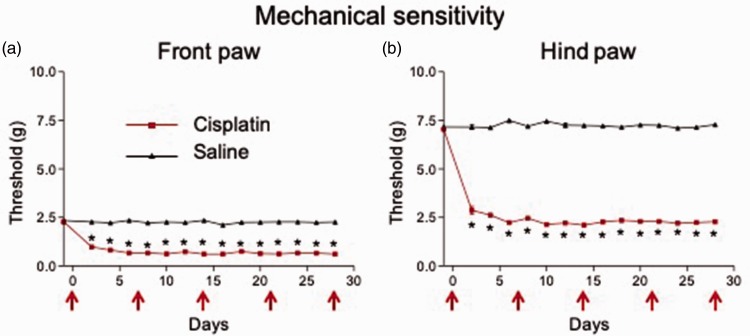
Weekly cisplatin injections result in stable mechanical hypersensitivity in the mouse front and hind paws.von Frey mechanical withdrawal thresholds were reduced two days after beginning weekly i.p. injections (red arrows) of cisplatin (n = 18 mice) in the front (a) and hind (b) paws compared to saline injected mice (n = 36 mice). Values remained consistent over a 28-day period, indicating the development of stable mechanical hypersensitivity. Means ± SEM are shown. *P < 0.0001 compared to saline; two-way ANOVA with Bonferroni posttests.

Cisplatin injection (i.p.; see “Cisplatin-induced neuropathy pain model” subsection in “Methods” section) resulted in a rapid (within two days), stable, and significant (n = 18 mice) reduction in von Frey withdrawal thresholds in the front (Figure 1(a); P < 0.0001, F_1,52_ = 844.0, repeated measures two-way ANOVA with Bonferroni posttests) and hind (Figure 1(b); P < 0.0001, F_1,  52_ = 6232, repeated measures two-way ANOVA with Bonferroni posttests) paws compared to saline (control) injection (n = 36 mice), indicating the development of mechanical allodynia.

### Ibuprofen and URB937 dose dependently inhibit cisplatin-induced mechanical hypersensitivity in the front and hind paws

**Figure 2.**
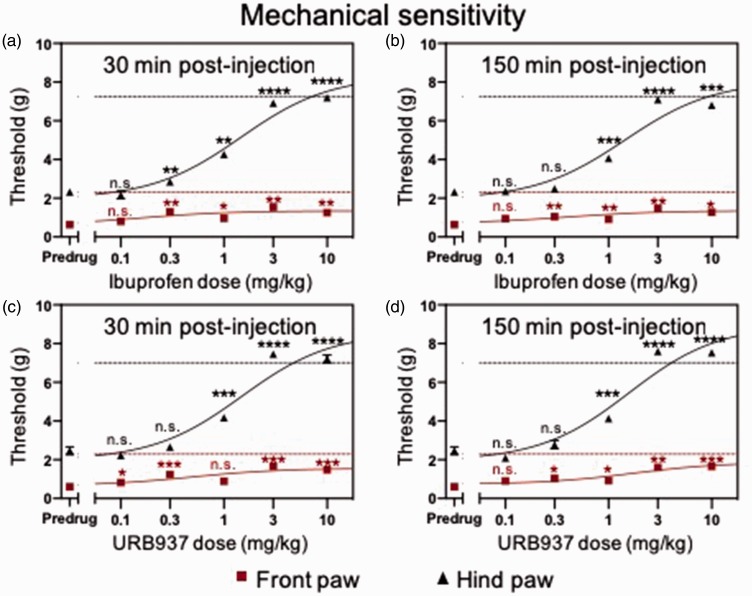
Ibuprofen and URB937 dose dependently inhibit cisplatin-induced mechanical hypersensitivity.Systemic injection of ibuprofen (a and b) or URB937 (c and d) dose dependently (0–10 mg/kg) increased von Frey withdrawal thresholds in cisplatin-treated mice (n = 5 mice (ibuprofen), n = 6 mice (URB937)) at 30 min (a and c) and 150 min (b and d) after drug administration. Dashed lines indicate normal (precisplatin) values. Means ± SEM are shown. *P<0.05, **P<0.01, ***, **** P<0.001 compared to predrug; repeated measures one-way ANOVA with Bonferroni posttests. Red lines refer to front paw and black lines refer to hind paw. n.s.: not significant.

Ibuprofen dose dependently (0.1, 0.3, 1, 3, and 10 mg/kg) inhibited von Frey withdrawal thresholds measured in the front and hind paws 30 min (Figure 2(a); n = 5 mice; P < 0.05, F_2.472, 19.78_ = 259.0, repeated measures two-way ANOVA with Bonferroni posttests; EC_50_ = 0.16 mg/kg (front paw); EC_50_ = 1.46 mg/kg (hind paw)) and 150 min (Figure 2(b); n = 5 mice; P < 0.05, F_2.245,  17.96_ = 302.0, repeated measures two-way ANOVA with Bonferroni posttests; EC_50_ = 0.41 mg/kg (front paw); EC_50_ = 1.44 mg/kg (hind paw)) after systemic (i.p.) injection compared to predrug values, indicating reduction in mechanical hypersensitivity associated with cisplatin-induced neuropathy.

Similarly, the peripherally restricted FAAH inhibitor URB937 dose dependently (0.1, 0.3, 1, 3, and 10 mg/kg) inhibited von Frey withdrawal thresholds measured in the front and hind paws 30 min (Figure 2(c); n = 6 mice; P < 0.05, F_3.169, 31.69_ = 327.8, repeated measures two-way ANOVA with Bonferroni posttests; EC_50_ = 0.49 mg/kg (front paw); EC_50_ = 1.49 mg/kg (hind paw)) and 150 min (Figure 2(d); n = 6 mice; P < 0.05, F_2.668,  26.68_ = 331.7, repeated measures two-way ANOVA with Bonferroni posttests; EC_50_ = 1.73 mg/kg (front paw); EC_50_ = 1.58 mg/kg (hind paw)) after systemic (i.p.) injection compared to predrug values, indicating reduction in mechanical hypersensitivity associated with cisplatin-induced neuropathy. We have previously reported that ibuprofen^17^ and URB937^18^ do not have effects on mechanical or thermal sensitivity in saline control-injected mice. Preliminary experiments (data not shown) showed that control saline injections did not have any effect on mechanical sensitivity.

### Differential front and hind paw mechanical responses following administration of Ibuprofen, URB937, Amitriptyline and Gabapentin

**Figure 3.**
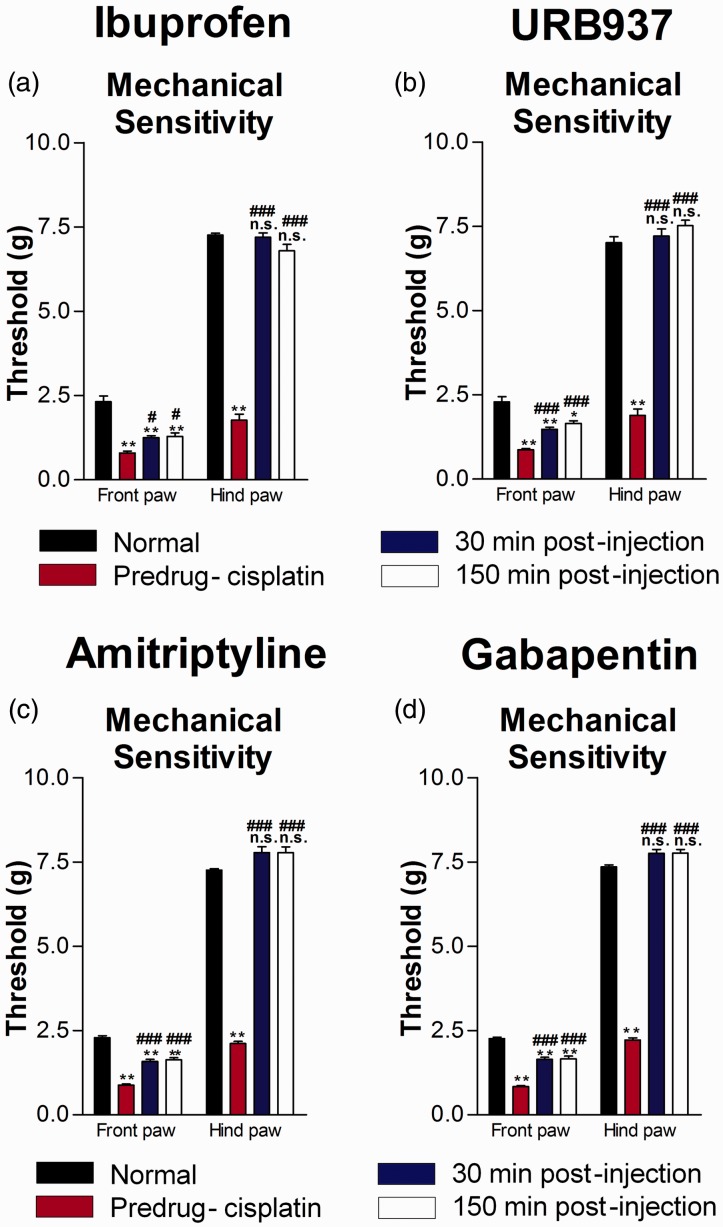
Ibuprofen, URB937, amitriptyline, and gabapentin result in complete normalization of mechanical hypersensitivity in the hind paw but not mechanical hypersensitivity in the front paw.von Frey mechanical withdrawal thresholds (a–d) were measured before initiation of cisplatin-induced neuropathy induction and before, 30 min, and 150 mins after i.p. injection of ibuprofen at 10 mg/kg (a; n = 5 mice) or URB937 at 10 mg/kg (b; n = 6 mice) or amitriptyline at 30 mg/kg (c; n = 6 mice) or gabapentin at 100 mg/kg (d; n = 6 mice). However, von Frey withdrawal thresholds returned to normal levels in the hind paw but not the front paw, suggesting that mechanical hypersensitivity was normalized in the hind paw but persisted in the front paw. Bar histograms show mean ± SEM. *P< 0.001, ** P<0.0001 compared to normal; ^#^P<0.05,^ ###^P<0.0001 compared to predrug; repeated measures one-way ANOVA with Bonferroni posttests. n.s.: not significant.

Systemic administration of ibuprofen (10 mg/kg) significantly attenuated mechanical sensitivity (Figure 3(a); n = 5 mice; P < 0.05, F = 35.1 (front paw), F = 329.7 (hind paw), one-way ANOVA with Bonferroni posttests) associated with cisplatin-induced neuropathy at 30 and 150 min after injection. However, von Frey withdrawal thresholds returned to normal levels only in the hind paws but not in the front paws. Similarly, for mechanical sensitivity, URB937 (Figure 3(b); n = 6 mice; P < 0.001, F = 39.9 (front paw), F = 219.3 (hind paw), one-way ANOVA with Bonferroni posttests), amitriptyline (Figure 3(c); n = 6 mice; P < 0.0001, F = 123.33 (front paw), F = 462.07 (hind paw), one-way ANOVA with Bonferroni posttests), or gabapentin (Figure 3(d); n = 6 mice; P < 0.0001, F = 103.99 (front paw), F = 861.60 (hind paw), one-way ANOVA with Bonferroni posttests) injections resulted in a return to normal levels only in the hind paws.

### Ibuprofen, URB937, amitriptyline, and gabapentin mechanical threshold in saline-treated mice

**Figure 4.**
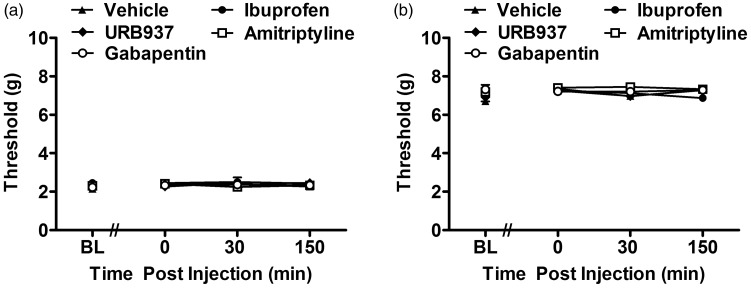
Ibuprofen, URB937, amitriptyline, and gabapentin failed to alter mechanical hypersensitivity in the front and hind paws of saline-treated mouse.von Frey mechanical withdrawal thresholds in the front (a) and hind (b) paws of saline-treated mice receiving either vehicle (n = 12 mice), ibuprofen (n = 6 mice), URB937 (n = 6 mice), amitriptyline (n = 6 mice), or gabapentin (n = 6 mice). No statistical difference was found in assessment of mechanical allodynia between vehicle relative to compound treatment groups for the front (P = 0.235) and hind (P = 0.104) paws. Means ± SEM are shown.

In saline-treated mice, ibuprofen, URB937, amitriptyline, and gabapentin failed to alter (n = 6–12 mice; F_4,  31_ = 1.47, P = 0.235 front paw, Figure 4(a); F_4, 31_ = 2.11, P = 0.104 hind paw, Figure 4(b)) mechanical withdrawal thresholds relative to vehicle treatment (Figure 4(a) and (b)) at any postinjection time point (F_12,  93_ = 1.37, P = 0.193, front paw, Figure 4(a); F_12, 93_ = 1.76, P = 0.068 hind paw, Figure 4(b)).

### Front relative to hind paws changes in cisplatin-induced cold sensitivity

**Figure 5.**
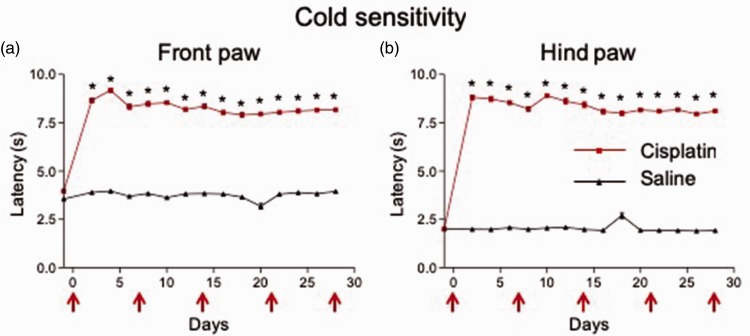
Weekly cisplatin injections result in stable thermal (cold) hyporesponsiveness in the mouse front and hind paws.Acetone cold responses were increased two days after beginning weekly i.p. injections (red arrows) of cisplatin (n = 18 mice) in the front (a) and hind (b) paws compared to saline injected mice (n = 36 mice). Values remained consistent over a 28-day period, indicating the development of stable decrease in thermal (cold) responsiveness, respectively. Means ± SEM are shown. *P < 0.0001 compared to saline; two-way ANOVA with Bonferroni posttests.

Cisplatin injection (i.p.; see “Cisplatin-induced neuropathy pain model” subsection in “Methods” section) resulted in a rapid (within two days), stable, and significant (n = 18 mice) increase in time until response to an acetone cold stimulus in the front (Figure 5(a); P < 0.0001, F_1,  52_ = 8720, repeated measures two-way ANOVA with Bonferroni posttests) and hind (Figure 5(b); P < 0.0001, F_1, 52_ = 42,850, repeated measures two-way ANOVA with Bonferroni posttests) paws compared to saline injection (n = 36 mice), indicating the development of cold hyposensitivity with cisplatin injection.

### Ibuprofen and URB937 dose dependently inhibit cisplatin-induced cold hyposensitivity in the front and hind paws

**Figure 6.**
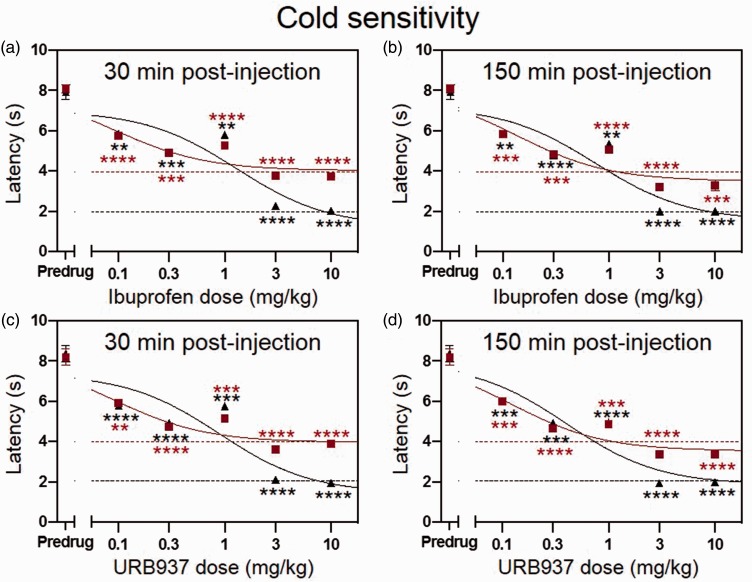
Ibuprofen and URB937 dose dependently inhibit cisplatin-induced thermal (cold) hyposensitivity.Systemic injection of ibuprofen (a and b) or URB937 (c and d) dose dependently (0–10 mg/kg) decreased acetone cold responses in cisplatin-treated mice (n = 5 mice (ibuprofen), n = 6 mice (URB937)) at 30 min (a and c) and 150 min (b and d) after drug administration. Dashed lines indicate normal (precisplatin) values. Means ± SEM are shown. **P<0.05, ***P<0.01, ****P<0.001 compared to predrug; repeated measures one-way ANOVA with Bonferroni posttests. Red lines refer to front paw and black lines refer to hind paw.

Ibuprofen dose dependently (0.1, 0.3, 1, 3, and 10 mg/kg) decreased the cisplatin-induced elevation in withdrawal latency to an acetone cold stimulus in the front and hind paws at 30 min (Figure 6(a); n = 5 mice; P < 0.01, F_3.403, 27.23_ = 802.4, repeated measures two-way ANOVA with Bonferroni posttests; EC_50_ = 0.09 mg/kg (front paw); EC_50_ = 1.30 mg/kg (hind paw)) and 150 min (Figure 6(b); n = 5 mice; P < 0.01, F_2.853, 22.82_ =748.7, repeated measures two-way ANOVA with Bonferroni posttests; EC_50_ = 0.14 mg/kg (front paw); EC_50_ = 0.75 mg/kg (hind paw)) postinjection compared to predrug values, indicating inhibition of cisplatin-induced cold sensitivity.

Similarly, the peripherally restricted FAAH inhibitor URB937 dose dependently (0.1, 0.3, 1, 3, and 10 mg/kg) decreased the elevated withdrawal latency to a cold acetone stimulus associated with cisplatin-induced neuropathy in the front and hind paws at 30 min (Figure 6(c); n = 6 mice; P < 0.0001, F_2.194,  21.94_ = 874.0, repeated measures two-way ANOVA with Bonferroni posttests; EC_50_ = 0.09 mg/kg (front paw); EC_50_ = 0.89 mg/kg (hind paw)) and 150 min (Figure 6(d); n = 6 mice; P < 0.001, F_2.730,  27.30_ = 791.5, repeated measures two-way ANOVA with Bonferroni posttests; EC_50_ = 0.12 mg/kg (front paw); EC_50_ = 0.40 mg/kg (hind paw)) postinjection compared to predrug values, indicating inhibition of cisplatin-induced cold sensitivity. Preliminary experiments (data not shown) showed that control saline injections did not have any effect on cold sensitivity.

### Similar front and hind paw cold responses following administration of Ibuprofen, URB937, Amitriptyline and Gabapentin

**Figure 7.**
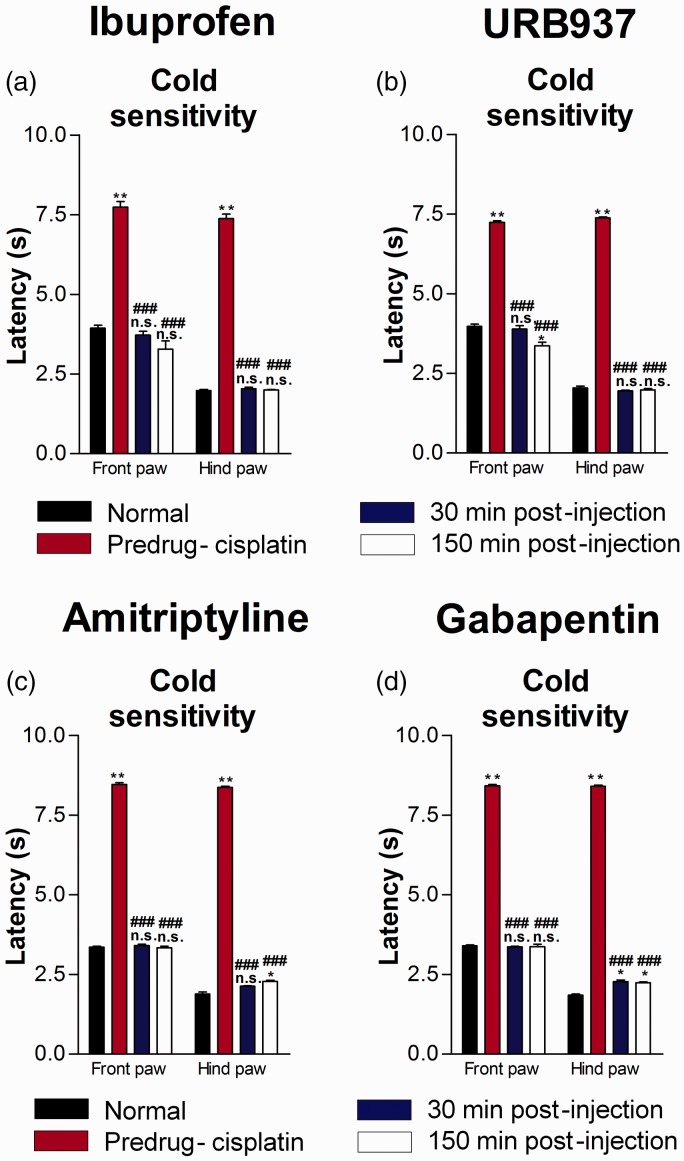
Ibuprofen, URB937, amitriptyline, and gabapentin result in complete normalization of cold responsiveness in the front and hind paws.Acetone cold responses (a and b) were measured before initiation of cisplatin-induced neuropathy induction and before, 30 min, and 150 min after i.p. injection of ibuprofen at 10 mg/kg (a; n = 5 mice) or URB937 at 10 mg/kg (b; n = 6 mice) or amitriptyline at 30 mg/kg (c; n = 6 mice) or gabapentin at 100 mg/kg (d; n = 6 mice). Acetone responses returned to normal levels in both the front and hind paws, suggesting that cold responsiveness was completely normalized in both paws. Bar histograms show mean ± SEM. *P<0.001, **P<0.0001 compared to normal; ^###^P < 0.0001 compared to predrug; repeated measures one-way ANOVA with Bonferroni posttests. n.s.: not significant.

Systemic administration of ibuprofen (10 mg/kg) significantly decreased the elevated withdrawal latency to cold stimulation (Figure 7(a); n = 5 mice; P < 0.0001, F = 140.9 (front paw), F = 1225 (hind paw), one-way ANOVA with Bonferroni posttests) associated with cisplatin-induced neuropathy in the front and hind paws at 30 and 150 min after injection. Indeed, acetone withdrawal latencies returned to normal levels in both the front and hind paws. Similarly, for cold sensitivity, URB937 (Figure 7(b); n = 6 mice; P < 0.001, F = 368.5 (front paw), F = 4392 (hind paw), one-way ANOVA with Bonferroni posttests), amitriptyline (Figure 7(c); n = 6 mice; P < 0.0001, F = 2678.08 (front paw), F = 5544.03 (hind paw), one-way ANOVA with Bonferroni posttests), or gabapentin (Figure 7(d); n = 6 mice; P < 0.0001, F = 3291.64 (front paw), F = 6871.30 (hind paw), one-way ANOVA with Bonferroni posttests) injections reversed cisplatin-induced cold hyposensitivity with values returning to normal levels in both the front and hind paws.

### Ibuprofen, URB937, amitriptyline, and gabapentin withdrawal latency in saline-treated mice

**Figure 8.**
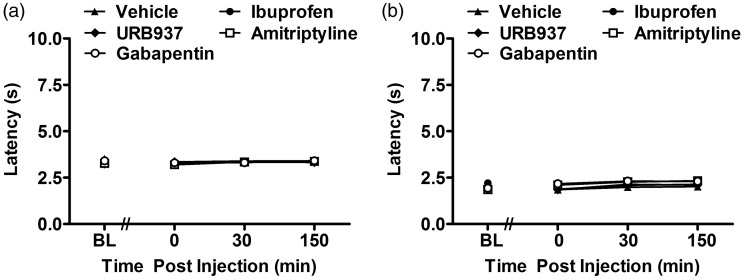
Ibuprofen, URB937, amitriptyline, and gabapentin failed to alter cold sensitivity in the front and hind paws of saline-treated mouse.Cold responsiveness to acetone in the front (a) and hind (b) paws of saline-treated mice receiving vehicle (n = 12 mice), ibuprofen (n = 6 mice), URB937 (n = 6 mice), amitriptyline (n = 6 mice), or gabapentin (n = 6 mice). No statistical difference was found in assessment of cold sensitivity between vehicle relative to compound treatment groups for the front (P = 0.188) and hind (P = 0.397) paws. Means ± SEM are shown. BL: baseline.

In saline-treated mice, ibuprofen, URB937, amitriptyline, and gabapentin failed to alter (n = 6–12 mice; F_4, 31_ = 1.65, P = 0.188 front paw, Figure 8(a); F_4, 31_ =1.05, P = 0.397 hind paw, Figure 8(b)) the frequency of withdrawal to acetone relative to vehicle treatment at any postinjection time point (F_12, 93_ = 1.35, P = 0.207, front paw, Figure 8(a); F_12, 93_ = 0.24, P = 0.996 hind paw, Figure 8(b)).

## Discussion

**Figure 9.**
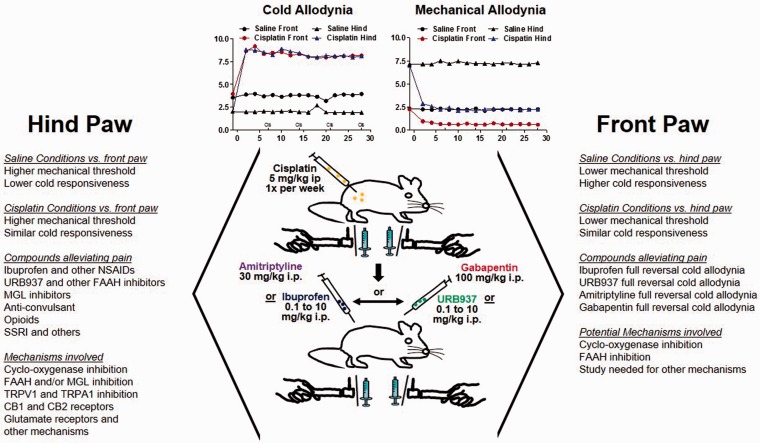
Graphical representation and comparison of front and hind paw pain-related findings associated with cisplatin-induced peripheral neuropathy.Findings for mechanical threshold and cold responsiveness at baseline and after induction of cisplatin-induced neuropathy were compared between the front and the hind paws. Antinociceptive drug responses in the front and hind paws and their associated mechanisms were also summarized. CB1: cannabinoid receptor 1; CB2: cannabinoid receptor 2; FAAH: fatty acid amide hydrolase; i.p.: intraperitoneally; MGL: monoacylglycerol lipase; NSAID: nonsteroidal anti-inflammatory drug; SSRI: selective serotonin reuptake inhibitor; TRPV1:transient receptor potential vanilloid 1; TRPA1: transient receptor potential ankyrin 1.

A key finding of this study is that rodent front paws exhibit mechanical hypersensitivity and thermal hyposensitivity following cisplatin administration. In addition, we found that the anticonvulsant (gabapentin), the antidepressant (amitriptyline), the NSAID ibuprofen, and the peripherally restricted FAAH inhibitor URB937 exhibit antinociceptive effects when pain behaviors are measured in the hind paws, but these beneficial effects are attenuated when measured in the front paws (Figure 9). To our knowledge, this is the first time that pain-related changes have been described in the front paws in a rodent cisplatin-induced neuropathy model and that differential antinociceptive responses have been identified between the front and hind paws.

Consistent with the previous studies,^21–23^ we found the development of mechanical hypersensitivity and thermal hyposensitivity in the hind paws in the cisplatin-induced neuropathy model. Interestingly, we found similar pain-related changes in the front paws (Figure 9). The mechanisms underlying these changes remain to be determined. Mitotoxicity is thought to be a significant pathophysiological mechanism for cisplatin-induced neuropathy.^3^ Cisplatin concentrations in the peripheral nervous system are similar to those in tumor tissue^3^ and cisplatin forms adducts with mitochondrial DNA in the dorsal root ganglion.^24–26^ Activation of the p38 mitogen-activated protein kinase (MAPK) and extracellular signal-regulated kinase 1/2 pathways as well as reduced c-Jun N-terminal kinase/stress-activated protein kinase phosphorylation has been implicated in the development of thermal hyposensitivity in cisplatin-induced neuropathy.^3^ Oxidative stress and free radical formation^27^ as well as CB_2_ receptor activation^21^ have also been associated with the pathophysiology of cisplatin-induced neuropathy.

URB937 is a peripherally restricted inhibitor of FAAH.^13,14^ URB937 inhibited writhing responses to acetic acid-induced visceral pain, mechanical, and thermal sensitivity induced by sciatic nerve ligation (SNL) and intraplantar carrageenan injection, respectively, and pain-related Fos activation in the spinal cord comparable to centrally acting FAAH inhibitors in a cannabinoid (CB) receptor 1-dependent mechanism.^13,20,28,29^ URB937 was superior to a centrally acting FAAH inhibitor on mechanical and thermal sensitivity from complete Freund’s adjuvant-induced arthritis pain and acted synergistically with the NSAID indomethacin to inhibit carrageenan and SNL-induced mechanical and thermal sensitivity.^20^ URB937 inhibited mechanical and thermal sensitivity in a cisplatin-induced neuropathy model of neuropathic pain in a CB_1_- and CB_2_-dependent mechanism.^23^ In a model of nitroglycerin-induced migraine pain, URB937 inhibited responses on the formalin test^30^ and reduced activation in the nucleus trigeminalis caudalis and the locus coeruleus.^31^ Furthermore, URB937 acted synergistically when administered with an inhibitor of soluble epoxide hydrolase in carrageenan-induced inflammatory pain and streptozocin-induced neuropathic pain on mechanical and thermal sensitivity.^29^ However, FAAH inhibitors have failed in clinical trials, with the centrally available FAAH inhibitor PF-04457845 failing to relieve osteoarthritis pain in human subjects.^32^ Similarly, NSAIDs such as ibuprofen have demonstrated preclinical efficacy for neuropathic pain;^33,34^ however, their clinical utility is limited.^35^

Similar to these preclinical studies, we found that systemic administration of URB937, ibuprofen, amitriptyline, and gabapentin significantly attenuated cisplatin-induced mechanical hypersensitivity and thermal hyposensitivity. Interestingly, this response differed by modality between the front and hind paws as mechanical hypersensitivity returned to normal levels in the hind paws but not in the front paws and thermal responsiveness returned to normal levels in both the front and hind paws. The mechanism(s) for these effects remain to be determined but are likely in the peripheral nervous system as the distribution of URB937 is peripherally restricted due to active exclusion from the central nervous system by the membrane transporter ABCG2.^36^ Cannabinoids^37^ and NSAIDs including ibuprofen^38^ have been reported to inhibit the MAPK pathway. Inhibition of cisplatin-induced elevations in MAPK activity could contribute to drug effects. Drug concentrations tested were in the plateau phase of the dose–response curve and peak effects on mechanical hypersensitivity were observed 30 min after injection, making it unlikely that drug dosage or the time point of measurements contributed to the lack of complete response in the front paws. It is possible that the higher number of mechanoreceptors present in the front paw may account for the differential mechanical sensitivity in the forepaws versus hind paws of mice after the systemic administration of URB937, ibuprofen, amitriptyline, and gabapentin. Mouse front paw glabrous skin is characterized by a density of mechanoreceptors three times higher than that of hind paw, which may explain why mechanical hypersensitivity levels returned to normal in the hind paws but not in the front paws.^39^

Many drugs that demonstrate preclinical efficacy as antinociceptive agents ultimately fail during clinical trials.^8,9^ Rodent front paws have different responses than the hind paws to neurological injury and have a greater degree of the fine sensorimotor functions that are characteristically damaged in patients with chemotherapy-induced neuropathy.^40–49^ Grooming behaviors in rodents demonstrate behavioral complexity and organization and primarily involve forepaw grooming of the face, head, neck, and trunk.^49^ Sensorimotor function of the forelimb is used to evaluate fine motor deficits following rodent cervical spine injury^45^ or peripheral nerve injury^48^ and correlates to tissue pathology. Paw reaching was significantly impaired in rats after a middle cerebral artery infarct, and this impairment was directly related to the lesion size.^44^ Furthermore, endothelin-1-induced ischemic damage to the anterior motor cortex consistently induced paw dragging behavior along the cylinder wall in the cylinder test instead of pushing off of the wall when moving from a rearing to a four-legged stance.^46,47^ In addition, forepaw fine motor grasping and reaching responses were impaired after manganese^41^ or prenatal lead^42^ exposure as well as fore and hind limb gait deficits.^42^ In a mouse model of Parkinson’s disease, forepaw sensorimotor function was impaired in the adhesive removal test.^43^ In addition, behaviors requiring forepaw sensorimotor function were consistently impaired on the adjusting steps test, challenging beam test, pole test, spontaneous activity test, and limb-use asymmetry test, whereas overall gait analysis was more variable. In a mouse model of Huntington’s disease, the gait swing time was affected in the forelimb but not in the hind limb in disease mice compared to control.^40^ Interestingly, the studies mentioned above demonstrate that cerebral artery infarcts or diseases such as Parkinson’s and Huntington’s cause impairment of forepaw sensorimotor function and therefore disturbance in forelimb sensitivity. It is surprising that pain studies using animal models have been focusing on the hind paws to assess disturbances in mechanical, cold, or heat sensitivity^13,16–18^ since fine motor functions are more developed in the front paws for rodents.^49^ Our study emphasizes the importance of evaluating analgesic effectiveness of compounds by thorough assessment of front paw sensitivity in conjunction with the hind paws.

Consistent with the idea of increased fine sensorimotor function in the front compared to the hind paws, we found that baseline and cisplatin-treated mechanical withdrawal thresholds were lower in the front paws than in the hind paws (Figure 9). Our findings corroborate with clinical data showing that chemotherapeutic treatments cause the development of chemotherapy-induced peripheral neuropathy in cancer patients which commonly manifests in the hands and feet with symptoms favoring sensory deficits.^50--52^ Therefore, our study strongly suggests that pain-related changes in the front paws show better predictive value for translation of preclinical findings in the context of chemotherapy-induced peripheral neuropathy.

## Conclusions

Ibuprofen, amitriptyline, gabapentin, and URB937 have beneficial effects on front and hind paw mechanical and cold sensitivity associated with cisplatin-induced neuropathy. However, pain behaviors return to normal levels in the hind paws but not the front paws in a modality-dependent manner. This suggests that measurement of front paw responses across multiple pain assays can provide reliable and accurate information about pain-related drug effects that might translate better to clinical findings. Future studies should be aimed at elucidating the mechanisms underlying these differential effects.
